# The future of theoretical evolutionary game theory

**DOI:** 10.1098/rstb.2021.0508

**Published:** 2023-05-08

**Authors:** Arne Traulsen, Nikoleta E. Glynatsi

**Affiliations:** ^1^ Department of Evolutionary Theory, Max Planck Institute for Evolutionary Biology, Plön 24306, Germany; ^2^ Max Planck Research Group: Dynamics of Social Behavior, Max Planck Institute for Evolutionary Biology, Plön 24306, Germany

**Keywords:** evolutionary game theory, bibliometric data, cooperation

## Abstract

Evolutionary game theory is a truly interdisciplinary subject that goes well beyond the limits of biology. Mathematical minds get hooked up in simple models for evolution and often gradually move into other parts of evolutionary biology or ecology. Social scientists realize how much they can learn from evolutionary thinking and gradually transfer insight that was originally generated in biology. Computer scientists can use their algorithms to explore a new field where machines not only learn from the environment, but also from each other. The breadth of the field and the focus on a few very popular issues, such as cooperation, comes at a price: several insights are re-discovered in different fields under different labels with different heroes and modelling traditions. For example, reciprocity or spatial structure are treated differently. Will we continue to develop things in parallel? Or can we converge to a single set of ideas, a single tradition and eventually a single software repository? Or will these fields continue to cross-fertilize each other, learning from each other and engaging in a constructive exchange between fields? Ultimately, the popularity of evolutionary game theory rests not only on its explanatory power, but also on the intuitive character of its models.

This article is part of the theme issue ‘Half a century of evolutionary games: a synthesis of theory, application and future directions’.

## 1. Why do we all fall in love with evolutionary game theory?

The problem of cooperation is ubiquitous in nature and society: why do animals give warning calls? While it increases the survival chances of their group, it comes with an increased individual risk which some individuals may choose to avoid. How do we distribute common tasks? In a shared apartment, somebody has to clean, but in the absence of clear rules, everybody is tempted to let others do the job. The theoretical framework to think about such strategic issues is game theory, which has transcended from its origins in mathematics into several disciplines in the past decades. Game theory today is used in the political and social sciences, in economics, and in psychology. Fifty years ago, Maynard Smith & Price [1] developed a beautiful approach to transfer game theoretical ideals to biology, an approach that ultimately led to a new field, evolutionary game theory. The history of this field is covered in this special issue by Parker [2].

The underlying game theoretic models for this kind of interaction are models that can be well explained and attract immediate attention. In addition, simple models for evolutionary dynamics were integrated into this system (often in the form of the replicator dynamics [3–5]). These evolutionary models appeal through their simplicity. However, also owing to their nonlinearities and their intuitive derivation, they represent popular examples of dynamical systems. In the past two decades, the theoretical interest in the field has been renewed, partly outside the field of biology. Especially in physics, the focus has shifted to the analysis of spatially extended systems [6] and stochastic evolutionary game dynamics [7].

For theoretical scientists coming from other fields, evolutionary game theory often presents a first contact with the world of evolutionary biology. Therefore, the field of evolutionary game theory also forms bridges with other disciples. For example, it naturally bridges from evolutionary biology to computer science and engineering, since computer simulations have always been embraced as a tool to understand evolutionary game dynamics in more complicated systems.

## 2. The current state of evolutionary game theory

Let us first try to summarize the current state of the field of evolutionary game theory. To avoid a subjective and possibly opinionated inventory, we performed a bibliometric analysis of papers published in the field.

### (a) A data analysis of the field of evolutionary game theory

We work with a bibliometric dataset collected from the German Competence Centre for Scientometrics (referred to as Kompetenzzentrum Bibliometrie (KB), a project for German science established in 2008 by the Federal Ministry for Education and Research: https://bibliometrie.info/en/) which has a quality controlled in-house data infrastructure based on the Web of Science (WoS). KB maintains several versions of the WoS database, each reflecting how the database changed over the years. We use the 2021 version which includes publications and citation counts up to the end of 2021. Publications on evolutionary game theory were matched by recording articles whose title, abstract or keywords contained at least one element from a set of predefined keywords. More information on the data collection can be found in appendix Ab.

The search criteria retrieved 7758 unique records dating from 1980 to 2021 (we can only retrieve publications from 1980 onwards from the database). The complete metadata for each publication include its title, the title of the publishing journal, the year of its publication, its references, and the list of authors which also includes their affiliations and their addresses. Moreover, the metadata also includes the records’ keywords and its research areas assigned by WoS.

We note that we did not retrieve any publications of John Maynard Smith, one of the founding fathers of this field. This is because many of his game theoretic publications were before 1980, and his later publications did not meet our search criteria. Nevertheless, by examining the list of references of each publication in the dataset, we obtained the most referenced published works and two of John Maynard Smith's are ranked amongst them (table 1). His book ‘*Evolution and the theory of* *games*’ [8] ranks first, and the original paper which introduced evolutionary game theory, ‘Logic of animal conflict’ ranks fourth [1]. This demonstrates the substantial influence that he has had on the field. The rest of table 1 includes publications from the early days of the field and from the late 1990s early 2000s. The latter publications are highlight the continued interest in populations with spatial structure.

**Table 1.** The scientific publications cited the most by the publications in our dataset. (These are the 10 scientific publications that appear the most in the list of references in our dataset. We take the list of references for each record in the data and count the number of times each of these references appears. The scientific articles and books in this table are not necessarily part of the dataset (appendix Ac). We can also examine which publications from our dataset are the most cited using their citation count from the WoS. This table can be found in appendix Ac.)

title	num. citations	authors	pub. year
Evolution and the theory of games	1666	Maynard Smith, John	1982
Evolutionary games and spatial chaos	1300	Nowak, Martin A and May, Robert M	1992
Evolutionary games on graphs	1054	Szabó, György and Fath, Gabor	2007
Logic of animal conflict	1004	Maynard Smith, John and Price, George R	1973
Five rules for the evolution of cooperation	964	Nowak, Martin A	2006
The evolution of cooperation	890	Sachs, Joel L; Mueller, Ulrich G; Wilcox, Thomas P and Bull, James J	2004
Evolutionarily stable strategies and game dynamics	888	Taylor, Peter D and Jonker, Leo B	1978
Scale-free networks provide a unifying framework for the emergence of cooperation	673	Santos, Francisco C and Pacheco, Jorge M	2005
Evolutionary Prisoner’s Dilemma game on a square lattice	619	Szabó, György and Töke, Csaba	1998
A simple rule for the evolution of cooperation on graphs and social networks	565	Ohtsuki, Hisashi; Hauert, Christoph; Lieberman, Erez and Nowak, Martin A	2006

### (b) Evolutionary game theory is scattered across disciplines

Evolutionary game theory is an interdisciplinary subject that has captivated researchers from a diverse list of scientific backgrounds and geographical locations. With our data, we can quantify this statement: 12 309 authors that have published in the field are captured by our dataset. For 9138 of these authors we have found a single publication, and for authors with multiple records we collected on average four. Authors in the field write from a large list of different countries, more specifically, from 87 countries with the majority of authors located in China (37.1%), the USA (16.5%), the UK (5.9%), Germany (3.6%), Japan (3.3%), Canada (2.8%) and France (2.8%).

As a proxy for authors’ disciplines, we use the research areas assigned to their publications. These research areas are collected by the WoS, and they are based on the journal a scientific work was published in (more details can be found in [9]). Essentially, we estimate an author’s discipline based on the journals where an author publishes the most. We count the number of publications in each research area for each author, and we assign to the author the one with the highest number of publications at the time of publication. In the case where an author has an equal number of publications in two (or more) research areas, we randomly allocate one to the author. The results indicate that authors come from a variety of disciples, more specifically, there are 122 research areas in the dataset, and the eight most representative are computer science (14.6%), engineering (9.8%), physics (9.8%), business and economics (8.0%), environmental sciences and ecology (7.5%), multidisciplinary sciences (7.0%), mathematics (6.5%), mathematical and computational biology (6.0%). We find it remarkable that actually only a small fraction of the papers in our database are in the area of biology (mathematical and computational biology, and partly environmental sciences and ecology), but this could arise from the different publication cultures in the different fields.

**Figure 1.**
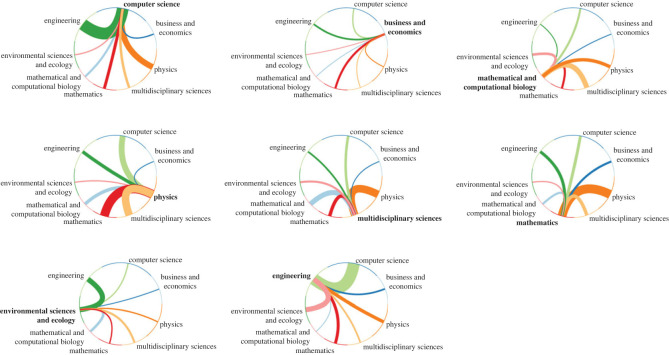
Interdisciplinary interactions between authors. The eight most prolific disciplines in the dataset are computer science, engineering, physics, environmental sciences and ecology, business and economics, multidisciplinary sciences, mathematics, and mathematical and computational biology. Each plot shows the connections of authors in one of these eight disciplines to authors from the others. Authors are represented as nodes and are sorted by discipline. An edge between two authors exists if and only if two authors have published together. The plot illustrates edges from one disciple to the others, this does not include in-group edges and neither connections to other disciplines. The width of the lines are equivalent to the number of connections. Thus, there are more mathematics authors connected to physicists compared to computer scientists. This figure shows not only that this is a highly interdisciplinary field, but also, for example, that there is a large body of scientific work in computer science and engineering that is only weakly connected to biology. Similarly, physicists and economists tend to work separately. (Online version in colour.)

We can evaluate the level of interdisciplinarity by combining the disciples of authors and the co-authorship network. Figure 1 gives a visual representation of interdisciplinary interactions, and it can be seen that authors from the eight most abundant disciples have a large amount of collaborations with each other. However, there are disciplines which are better connected than others: computer scientists are more likely to write with engineers compared to biologists, and physicists are better connected to mathematicians compared to economists. Regardless, figure 1 illustrates a tendency of interdisciplinary interactions between authors.

### (c) Cooperation is currently the focus of evolutionary game theory

**Figure 2.**
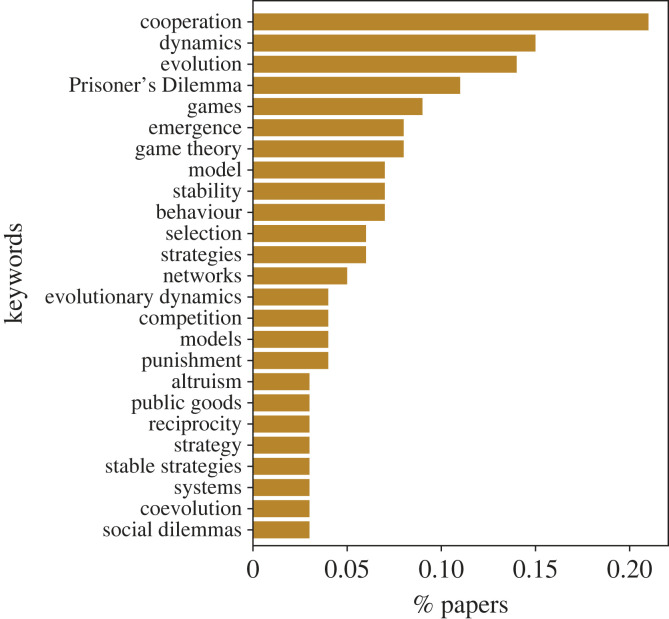
The most commonly used keywords. Keywords assigned to publications are a combination of authors’ keywords and systematic keywords. Systematic keywords are generated by the WoS from the titles of cited articles. There are a total of 20 700 unique keywords in the dataset. The 25 most commonly assigned keywords are shown in this bar plot, and more specifically, the percentage of papers that have used each keyword. A publication can have multiple keywords, and on average (in our data) a publication has eight keywords. For the data collection, a different set of keywords were used to search for relevant articles (see appendix Ab), these are omitted here. The most commonly used keyword, in approximately 20% of the publications, is the word ‘cooperation’ which verifies that there is a major focus of evolutionary game theory in cooperation. This is not the only word that displays this, other words include: the ‘Prisoner’s Dilemma’, ‘public goods’, ‘altruism’ and ‘reciprocity’. The keywords ‘stability’, ‘strategies’, and ‘stable strategies’ demonstrate an important subject in the field which is the evolutionary stability of strategies. Moreover, the key ‘networks’ demonstrates another popular subject which is the study of spatial structured populations. (Online version in colour.)

A major focus of evolutionary game theory is cooperation among selfish actors, and sometimes evolutionary game theory seems to be reduced to the evolution of cooperation. Also this can be quantified: we deliberately chose the search terms for our database such that they do not focus on this field, see appendix Ab. Nonetheless, we find that a large number of papers focus on cooperation, see figure 2. Cooperation is a topic that has been studied extensively and has been named one of the biggest open problems in science [10]. However, by now we have found many mechanisms that explain cooperation [11]. Maybe we should extend the focus towards other issues that can be addressed by evolutionary game theory, such as analyse complex interactions [12,13], cyclic dynamics [14–18] and also social interactions of multiple actors [19–22]. It can also be the basis to analyse more complex social issues such as polarization [23–25].

### (d) The mathematical developments in evolutionary game theory can sometimes be detached from the systems that are the focus of the application

Theoretical scientists are not always driven by applications, but often they are driven by the further development of modelling tools and the development of theories that summarize more general features. For example, the development of stochastic evolutionary game theory for non-spatial systems [7] has led to a huge number of new theoretical results and helped to build bridges into population genetics [26–30], but many of these developments were also mostly theoretical and mathematical [31,32] and are not of immediate relevance for applied researchers or those developing concrete models for concrete systems. The knowledge of certain fixation probabilities, an important quantity in stochastic evolutionary games, is of limited use when trying to understand what is going on in a behavioural experiment. Instead, the focus on fixation probabilities and random effect has unlocked a whole new set of modelling tools and theoretical approaches, such as, for example, the use of weak selection in evolutionary games [7,20,28,33–35] that will only gradually find their way into the more applied and experimental literature.

We should acknowledge where results are mostly theoretical and emphasize that any such result would not immediately be crucial for real-world applications. For instance, new models for the evolution of cooperation can be helpful and interesting, but many of these papers start out arguing that they will have an immediate impact on human societies.

### (e) Experimental challenges are often not appreciated by theoretical scientists

Evolutionary game theory rests on a very small set of simple assumptions: players interacting in a game can be successful to a different degree. If strategies are copied by others (or inherited) based on the success of these strategies, then we can describe the dynamics in terms of evolutionary game theory. However, we need to be careful in the comparison between experiment and simulation. Based on behavioural experiments, some theoretical scientists have argued that the imitation dynamics in humans is quite different [36,37] compared to what the models assume, and the outcomes are different—cooperation is not promoted in the way that the models predict.

Recently, some theoretical scientists have accepted the challenge to run their own behavioural experiments [36,38–40]. As a prerequisite, they had to make themselves familiar with experimental design, which is largely neglected in the theoretical sciences. Often, these behavioural experiments call for a much closer investigation and lead to many new interesting research directions themselves—also outside of evolutionary game theory. On the other hand, theoretical researchers should carefully analyse what they can learn from such experiments. For example, a lot of theoretical research has focused on the case of low mutation rates, where new strategies are rarely introduced and most of the time, the population is dominated by a single strategy [41–43]. This approach allows us to look at the average abundances of strategies even in complex settings with multiple strategies, as it reduces the dynamics from a state space that captures on the order of *N*^*d*^ states (where *N* is the number of players and *d* is the number of strategies) to an embedded Markov chain with only *d* states. This approach is very useful to analyse games with many strategies and many possible equilibria for the associated deterministic dynamics [34,44,45]. However, does this already give a meaningful prediction for what happens in behavioural experiment of game theory? It is known that humans update their strategy in a very different way, as discussed by Grujić and co-workers [46,47]. Since one cannot expect that the average abundance of the strategy—especially in complex games with many strategies—is independent of the dynamics, thus one should not just compare e.g. the average level of cooperation in an experiment to a simulation without making a comparison of the underlying dynamics as well.

### (f) Theoretical challenges are often not appreciated by experimental scientists

Experimentalists in biology often ask to develop detailed models that take many aspects into account and that are ideally at the same time ‘predictive’. To set up such a model is usually not a major challenge, but these complex models can be analysed only by extensive simulations and they typically do not allow detailed understanding or to make connections to other fields and other phenomena.

An important issue is also that there are many ways of setting up a model or a simulation. In particular in finite populations and when the populations are structured, many additional assumptions form the basis of a model—for example, the exact way in which individuals copy others (e.g. through an imitation function or revision protocol [48]) or the exact properties of the population structure [6,49]. Also the choice of evolutionary dynamics can be crucial and the fields have different preferences: for biological models, a lot of scientists focus on models which have a parameter that controls the intensity of selection and thus the tendency to increase fitness or not [7,50]. By contrast, many simulations in computer science prefer to replace e.g. the worst 10% of the population with offspring of the best 10% of the population [51], which corresponds to strong selection in biology.

Many aspects of evolutionary game dynamics require the development of theoretical tools that are shaped by mathematical approaches and do not immediately drive any concrete insights on any applied problem—such work is usually not appreciated by experimentalists. For example, one of the most beautiful results in mathematical biology is the equivalence between the replicator dynamics for matrix games and the Lotka–Volterra equations [52]. This ingenious result may be of limited use for people interested in applying either ecological models or game theoretical models, but it is of tremendous value for those developing the theory and aiming to understand the general features of the dynamics.

If the goal of a model is prediction instead of understanding, other tools can be more appropriate. In this case, also machine learning approaches can then be applied if a sufficient amount of data are available—and the abstract models typically used in evolutionary game theory could be less relevant.

### (g) Many aspects are mostly relevant for human behaviour, calling for a closer interaction with social scientists

Behavioural ecology provides a wide range of fascinating applications for evolutionary game theory [53,54]. Even sophisticated social behaviour between species can be found in animals, with reputation effects in cleaner fish maybe one of the most celebrated examples [55]. Nonetheless, many of the more sophisticated models are developed with human behaviour in mind. This development calls for a much closer interaction with social scientists and economists, who often have a very different perspective on human behaviour. A key question is then which insights or novel approaches evolutionary game theory can provide for these fields and whether the aspects of behaviour captured by the evolutionary game are so far not in the focus of these fields. Without such interactions with scientists interested in very similar issues, it is less likely that something relevant and new emerges.

Also other areas of evolutionary game theory develop largely in parallel: for example, in computer science ‘the price of anarchy’ is discussed as the cost that individual optimization causes compared to external enforcement of a social optimum [56,57]. In evolutionary biology, external enforcement of optimal solutions is usually not even discussed, as individual selection is blind to such social optima. Thus, the focus is not on an efficiency comparison between different solutions, but on the possibility to evolve or stabilize cooperative solutions. These two problems are conceptually very similar, but the fields largely develop on their own.

### (h) Many aspects of biological evolution do not require game theory

A holistic understanding of many biological systems requires us to take into account many aspects, but game theory is not always the best starting point. For example, the ecology and evolution of cancer is complex and can only be fully understood if we take frequency and density-dependent aspects into account [58–61]. On the other hand, our understanding for the evolution of cancer has been tremendously advanced by genomics, which is typically based on a family of models where fitness effects are fixed (or even absent) and not frequency dependent [62–65]. Also, our understanding of microbial communities does often not explicitly require game theoretical interactions [66,67]. If such ‘simpler’ models are sufficient to understand the key features of a biological system, a game theoretical model with the many associated parameters may be an overkill—driven by the desire to apply a class of nice models, but less so by the system at hand.

## 3. The future

While this article is supposed to be about the future, we spent a lot of space writing about the present state of the field of theoretical evolutionary games. We can expect that also evolutionary game theory is subject to historical contingency—its future will be strongly dependent on where we are coming from. Thus, it seemed important to recapitulate where we are.

Moreover, any statement about the future of a scientific field may be pure speculation and here we can only mention a few issues that may deserve closer attention and that are already now on the horizon.

### (a) Advances in terms of the game and in terms of the dynamics

It seems to be important to distinguish two separate issues here: there are new and exciting games to study, but often it is sufficient to study them using established modelling tools. Some of these tools will probably remain important in the future. For example, it is unlikely that we will ever stop using the replicator dynamics as one of the most powerful approaches. However, the toolset in terms of the underlying dynamics is constantly extended. One example is the focus on stochastic evolutionary game dynamics in the past two decades [7]. It is unclear what advances will be made in terms of dynamics, in particular in terms of models that are analytically accessible, but it is likely the case that new dynamical models will be developed and established.

Other advances concern the games considered and the strategies taken into account. One example is the unexpected discovery of new strategies in repeated games that can induce their interaction partners to cooperate. This development of the theory was remarkable, as it came from ‘newcomers’ in the field of evolutionary game theory [68]. In addition, more and more situations are described by evolutionary game theory, from the social dilemmas arising from fair resource consumption in the face of climate change [69] to social dilemmas arising in the COVID-19 pandemics [70].

### (b) A common language

Evolutionary game theory papers are sometimes laden with field specific terms starting from [1, p. 16], who wrote ‘Therefore natural selection will cause alleles for [·· ·] behaviour to increase in frequency’. Some of these terms may appear to be confusing for physicists. However, also physicists come with their own terms e.g. [6, p. 127] write in their excellent review ‘Notice that for potential games this formula is analogous to the (negative) energy of an Ising-type model’. This kind of language does not make it easy to read such papers for scientists, e.g. from computer science. Computer scientists like to refer to terms like ‘empirical’ and ‘numerical experiments’ when they refer to individual-based simulations—terms that may be confusing for the biologists.

We hope that it will become common practice to clearly define the terms in a paper, and to stop relying on the reader’s knowledge of ‘the literature’.

### (c) Strategy sets

In their classical 1973 paper, Smith & Price [1] hand-picked five quite special strategies to illustrate an important point: limited war strategies can be successful. Ever since, in the case of discrete games people have chosen to analyse only those strategies they are interested in.

The choice of a strategy set seems quite arbitrary in the literature. We aim to explain the evolution of some behaviour and include it in the model. However, this may lead to a bias, as we cannot explain the presence or absence of a behaviour that we have not included in the model in the first place. For example, a model that explains the emergence of pro-social punishment [71] cannot explain the absence of anti-social punishment if such strategies were not taken into account in the first place [45]. To move beyond such complications, one has to choose the strategy set very carefully—an algorithmic definition that for example includes all memory one strategies in a Prisoner’s Dilemma seems more meaningful than the *a priori* choice of certain ‘interesting’ strategies, making the associated results much more robust [72].

In the traditional approach to evolutionary game theory based on the replicator dynamics, including additional strategies often does not change anything, in particular if they are strictly inferior to a strategy already present. However, in the stochastic case, especially under weak selection, this can have a huge influence, as even dominated strategies can be decisive for the long term outcome [44,73]. Therefore, conventional wisdom—such as the approach of iterated removal of dominated strategies—has to be re-visited when new dynamics are introduced.

### (d) Evolutionary game theory and data

Developing the theory of evolutionary games has been mostly driven from the mathematical and computational side, but there have always been connections to biological data and behavioural experiments. However, typically the data is derived from equilibrium states and much less is known about dynamics. Moreover, ideally we do not just collect data and look at the correlations within it, but we perform controlled experiments with well designed controls. This typically requires a much bigger effort, though. However, in many cases, such as in long-term evolution, experiments are not even possible—which makes theoretical models even more important.

A central goal in biology is to understand behaviour in the natural habitat. Taking humans into a laboratory at a university is thus problematic in many aspects: Participants in these studies are typically white, educated, industrialized, rich, and developed [74]—and cross cultural experiment reveal a lot of diversity [75,76]. Moreover, humans are not studied in their natural surroundings. An escape out of this are hidden experiments in everyday life [77] or adding behavioural experiments to popular computer games [78].

In addition, we anticipate that the future may bring entirely new kinds of data, for example the interaction between artificial intelligences.

### (e) The interaction of artificial intelligences as a new frontier

Game theory was brought into biology to understand the interactions between animals. Later, an approach was added that allowed us to understand populations of animals, where their behaviour is not static but can change over time, leading to evolutionary game theory in the modern sense, which can also be applied to bacteria and virus populations. Currently, there is a lot of interest in the interaction between humans, but the decision processes in humans may be quite different from those we usually use in our models. A new frontier where the decision processes are actually clearer is machine learning and artificial intelligence [79]. So far, these algorithms are usually trained by fixed training sets which are obtained in a situation where there are no other artificial intelligences. With these technologies pervading our lives more and more, we will soon run into situations where artificial intelligences interact with each other. In the case of the simplest kind of single layer neural networks, such interactions can be understood analytically [80], but interactions between deep neural networks make numerical approaches necessary. This naturally leads to many applications: with self-driving cars interacting on our roads or artificial assistants negotiating appointments with each other, a new playing field will be available for game theoretical approaches. Many of the traditional problems of game theory, such as equilibrium selection or the maintenance of cooperation, will continue to be relevant, but now the dynamics to approach such equilibria and to switch between them may be completely different.

### (f) Reproducibility of theoretical work

One important aspect of scientific work is reproducibility. There is currently a large push to make data and computer code accessible online with scientific publications. This is mainly to avoid yet another reproducibility crisis, but also to improve the overall quality of research and to remove any barriers from replicating, reproducing or building on existing findings.

In contrast to some common beliefs, even in the purely theoretical world reproducibility is not always given. Mathematicians and physicists writing statements such as 'some algebra shows’ can cause a lot of unnecessary work and in the worst case, their proofs may even be wrong or incomplete, as Fermat’s famous sentence ‘I have discovered a truly marvellous proof of this, which this margin is too narrow to contain’ reveals [81, p. 96]. The more immediate problem is reproducibility of numerical work, though.

The problem of reproducibility starts at making data available. Researchers for far too long had little to no incentive to publish their data in an accessible form. A closely related issue with numerical work is software/code. There are many situations where the main contribution from the research is not simply the underlying data, but the software used to produce the findings or conclusions. Sharing software can be as critical as sharing data, whether the code was used for simulations or for analysis. There are many examples of authors writing statements such as ‘Reasonable requests for computer code should be addressed to the corresponding authors’. In such case, it remains unclear what a ‘reasonable request’ would be and if it is appropriate to maintain such barriers to reproducibility. In evolutionary game theory, the importance of sharing data and code has been discussed for a long time, nevertheless authors in our field also fall short. We manually reviewed the 10 most referenced publications by our dataset with a publication day after 2017, which either explored experimental data or generated them using computer simulations (see table 3 and appendix Ac). Only two of these papers had made their data accessible [82,83].

In addition to making the data available, it is important to provide clear documentation of what exactly the data is, how they were generated, processed and analysed.

We realize that these practices are not trivial. Depending on the data source, making data available can be tricky (this work itself is an example of that), and maintaining software or even knowing how to document and to make software installable for others are skills that researchers are not necessarily trained to have. We anticipate that there will be more opportunities for researchers to learn about these practices in the future. Currently, the gold standard is to at least share documented data (e.g. via Zenodo) and code (e.g. via GitHub). We anticipate that in the future, such standards will become more and more common in the community, even if the additional work leads to a decrease in productivity.

### (g) Evolutionary game theory and behavioural ecology

There are many parts of theoretical evolutionary game dynamics that we have not touched upon, such as games with continuous strategies and G-functions (e.g. [84]). Also adaptive dynamics is an essential part of evolutionary game theory, as in this case one looks at long term evolution in a situation of frequency dependence [85–90]. It is particularly powerful as it allows us to analyse solutions that would naturally evolve and thus compare them with behaviour observed in ecology. There are a large number of fascinating applications of evolutionary game theory to behavioural ecology, from the classic example of blood sharing in vampire bats [91] to evolutionary games between microbes [92]. We anticipate that experimental biology will continue to be inspired by this field and find additional examples of game theoretical interactions in nature.

However, we focused here more on the theoretical developments in evolutionary game theory, as this field has moved way beyond its origins in evolutionary biology. As our literature analysis above shows, by now the majority of the scientific papers in evolutionary game theory is written outside biology and the field has taken on a life of its own. Many of these developments may be tangential to biological problems, but we feel it is important to acknowledge that the field has branched out into many other areas by now.

### (h) A note on gender

Two recent bibliometric studies, on the Prisoner’s Dilemma game [93] and on the field of cultural evolution [94], have shown that in large publication datasets, the most central authors based on co-authorship are male. This also holds for our dataset where the most central authors are male (appendix Ad). However, there are many fundamental contributions in the field driven by women (e.g. [36,60,95–101]).

Many researchers, in particular women, have used game theory as a stepping stone into related fields such as biology and ecology. We consider this to be a great feature of the field of evolutionary game theory. Regardless, one should point out that for newcomers looking for role models in their field, a list of central authors consisting of only white males can be intimidating.

## 4. Conclusion

Evolutionary game theory is a fascinating framework to model the evolutionary dynamics of interacting agents with many applications across disciplines. In the future, we hope for a tighter interaction between these disciplines, in particular as new fascinating applications are appearing these days. We are convinced that the next 50 years of evolutionary game theory will be at least as exciting as the past 50 years!

## Figures and Tables

**Figure 3.**
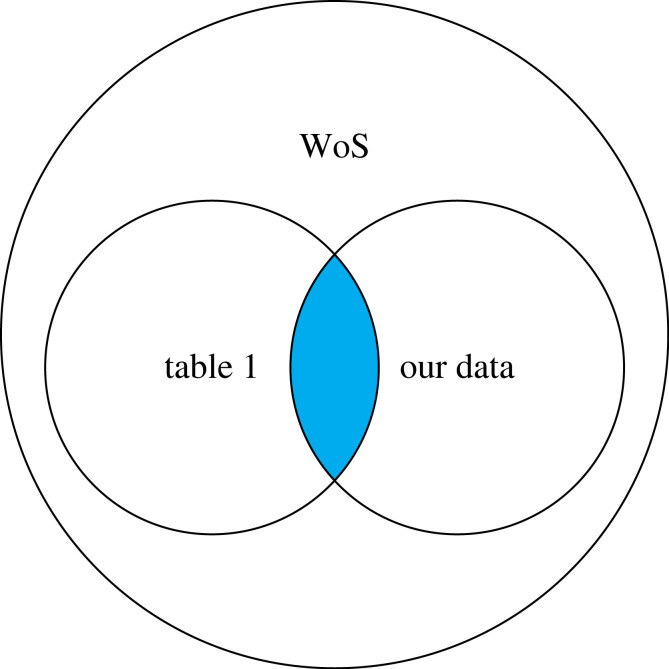
The relationship of the sets (1) publications in the WoS (2) publications of table 1 (3) publications in our dataset. All publications presented here are records in the WoS database. The data collection we conducted yielded 7758 publications on evolutionary game theory. Each of these publications cites a number of other scientific works which includes works on evolutionarily game theory and not. Not all evolutionary game theoretic works met our research criteria and so table 1 includes items from the WoS but not necessarily from the data. (Online version in colour.)

## Data Availability

We accessed the WoS data thanks to the Competence Centre for Bibliometrics (Kompetenzzentrum fr Bibliometrie). Our contractual agreement precludes us from redistributing the raw unprocessed data (i.e. the individual-level publication records). Users interested in accessing the microdata should contact the Web of Science or the Kompetenz-zentrum fr Bibliometrie directly to enquire about the conditions of access and use (https://www.bibliometrie.info/index.php?id=kontakt). The code for cleaning and analysing the data, as well as creating the figures presented here are available online: http://gitlab.evolbio.mpg.de/traulsen/philtrans.

## Appendix A

**(a) Data availability**

We accessed the WoS data thanks to the Competence Centre for Bibliometrics (Kompetenzzentrum für Bibliometrie). Our contractual agreement precludes us from redistributing the raw unprocessed data (i.e. the individual-level publication records). Users interested in accessing the microdata should contact the Web of Science or the Kompetenz-zentrum für Bibliometrie directly to enquire about the conditions of access and use (https://www.bibliometrie.info/index.php?id=kontakt).

Regardless, we aim to share as much as possible. The code for cleaning and analysing the data, as well as creating the figures presented here are available online: http://gitlab.evolbio.mpg.de/traulsen/philtrans. We carried out the analysis using Jupyter Notebooks. In each notebook, we have included comments/discussion documenting each step.

**(b) Data collection**

All articles used in this study were retrieved from the WoS via KB. Publications on evolutionary game theory were matched by recording articles whose title, abstract or keywords contained at least one element from a set of predefined keywords. The search terms used in the title and abstract fields were the following:

— ‘evolutionary game’
— ‘evolutionary game theory’
— ‘replicator dynamics’
— ‘moran process’
— ‘evolutionary stable strategies’
— ‘evolutionary game dynamics’
— ‘evolutionary stable strategy’
— ‘evolutionary’
— ‘Prisoner’s Dilemma’
— ‘evolutionary public goods’
— ‘evolutionary Hawk-Dove’
— ‘evolutionary Hawk Dove’
— ‘war of attrition’
— ‘evolutionary sex ratio game’
— ‘population games’
— ‘population game’

The search terms used for the keyword field were,

— ‘evolution of cooperation’
— ‘evolutionarily stable strategy’
— ‘evolutionary stable strategies’
— ‘evolutionary game’
— ‘evolutionary games’
— ‘evolutionary game dynamics’
— ‘evolutionary game theory’

The complete metadata for each resulting publication that were collected include metadata such as the title, the name of the publishing journal, the year of publication, the authors’ full names, their respective affiliations and addresses of their affiliations, the keyword associated with each article, the research areas and references.

Following the data collection we performed a data cleaning process where we identified and excluded any irrelevant articles. Furthermore, we manually checked and standardized the keywords. For example ‘Prisoner’s Dilemma game’ was replaced with ‘Prisoner’s Dilemma’. The names of the authors were also manually checked and standardized. The full name of an author includes their last name, full first name if given otherwise first initial, and the first initial of the second name. For example, entries ‘Santos, Francisco’ and ‘Santos, FC’ were replaced with ‘Santos, Francisco C’. We also checked all entries including special characters, for example ‘Wang, Bing-Hong’ was replaced with ‘Wang, Bing Hong’.

**(c) Most referenced and most cited scientific publications**

For table 1, we consider the list of references of each record in the dataset. We count the number of times each of the references is used by a work that we have collected. Not all the references are publications that meet our search criteria. Moreover, we did not manually add the references in the dataset since publications cite work that is not in evolutionary game theory. Thus, table 1 includes publications that are not part of the dataset and the dataset includes some works that are shown in table 1 but not all. A diagrammatic representation of this relationship is shown in figure 3.

For each record in the dataset, we can collect the number of citations that the record has. This count includes the overall citations a publication has received since its publication. The most cited publications from our data are shown in table 2.

**Table 2.** The publications from our dataset with the most citations. (The number of citations considers all the citations a publication has received. In this table, we do not constraint them to be from works that are in the dataset. The number of citations is collected from the WoS. As a reminder, we are using a copy of the 2021 WoS database. Thus, the number includes all the citations up to 2021.)

title	authors	num. citations	pub. year
Evolutionary games on graphs	Szabó, György and Fath, Gabor	1748	2007
The structure and dynamics of multilayer networks	Boccaletti, Stefano; Bianconi, Ginestra; Criado, Regino; Del Genio, Charo I; Gómez-Gardenes, Jesús; Romance, Miguel; Sendina-Nadal, Irene; Wang, Zhen and Zanin, Massimiliano	1382	2014
Coevolutionary games-a mini review	Perc, Matjaž and Szolnoki, Attila	1183	2010
Scale-free networks provide a unifying framework for the emergence of cooperation	Santos, Francisco C and Pacheco, Jorge M	1055	2005
Spatial structure often inhibits the evolution of cooperation in the snowdrift game	Hauert, Christoph and Doebeli, Michael	932	2004
Learning, mutation, and long-run equilibria in games	Kandori, Michihiro; Mailath, George J and Rob, Rafael	921	1993
Evolutionary Prisoner’s Dilemma game on a square lattice	Szabó, György and Tőke, Csaba	920	1998
Domestic political audiences and the escalation of international disputes	Fearon, James D	917	1994
The rock-paper-scissors game and the evolution of alternative male strategies	Sinervo, Barry and Lively, Curt M	902	1996
A strategy of win stay, lose shift that outperforms tit-for-tat in the Prisoners-Dilemma game	Nowak, Martin and Sigmund, Karl	847	1993
Social diversity promotes the emergence of cooperation in public goods games	Santos, Francisco C; Santos, Marta D and Pacheco, Jorge M	801	2008
How should we define fitness for general ecological scenarios	Metz, Johan AJ; Nisbet, Roger M and Geritz, Stefan AH	747	1992
The behavioural ecology of personality: consistent individual differences from an adaptive perspective	Dall, Sasha RX; Houston, Alasdair I and McNamara, John M	746	2004
Emergence of cooperation and evolutionary stability in finite populations	Nowak, Martin A; Sasaki, Akira; Taylor, Christine and Fudenberg, Drew	744	2004
Evolutionary dynamics of group interactions on structured populations: a review	Perc, Matjaž; Gómez-Gardenes, Jesús; Szolnoki, Attila; Floría, Luis M and Moreno, Yamir	716	2013
Emergence of cooperation and organization in an evolutionary game	Challet, Damien and Zhang, Yi-Chen	707	1997
The dynamical theory of coevolution: a derivation from stochastic ecological processes	Dieckmann, Ulf and Law, Richard	665	1996
The structural basis of protein folding and its links with human disease	Dobson, Christopher M	658	2001
Evolutionary dynamics on graphs	Lieberman, Erez; Hauert, Christoph and Nowak, Martin A	653	2005
Why are stabilizations delayed	Alesina, Alberto F and Drazen, Allan	634	1991
Evolutionary dynamics of biological games	Nowak, Martin A and Sigmund, Karl	611	2004

**(d) Central authors**

We construct the co-authorship network where the nodes of the network represent authors and an edge connects two authors if and only if those authors have written together. There are 12 309 authors in our dataset and thus the network has 12 309 nodes, and it also has 22 949 edges. We evaluate the most connected authors in the data based on centrality measures. We report four different centrality measures, namely, the degree, the closeness, the betweenness and the eigenvector centralities (tables 3 and 4).

**Table 3.** The scientific works cited the most by the publications in our dataset with a publication date after 2017. (Similarly to table 1, we consider the most referenced scientific works. We have manually evaluated the articles listed here. More specifically, we checked whether the data and/or code used in the manuscript were made available. Eight out of these 10 articles did not make either available.)

title	authors	num. citations	pub year
Punishment diminishes the benefits of network reciprocity in social dilemma experiments	Li, Xuelong; Jusup, Marko; Wang, Zhen; Li, Huijia; Shi, Lei; Podobnik, B; Stanley, H. Eugene; Havlin, Shlomo and Boccaletti, Stefano	52	2017
Using evolutionary game theory to study governments and manufacturers’ behavioral strategies under various carbon taxes and subsidies	Chen, Wanting and Hu, Zhi-Hua	47	2018
Exploiting a cognitive bias promotes cooperation in social dilemma experiments	Wang, Zhen; Jusup, Marko; Shi, Lei; Lee, Joung-Hun; Iwasa, Yoh and Boccaletti, Stefano	44	2018
Scaling the phase-planes of social dilemma strengths shows game class changes *n* the five rules governing the evolution of cooperation	Ito, Hiromu and Tanimoto, Jun.	43	2018
Promoting cooperation by punishing minority	Yang, Han-Xin and Chen, Xiaojie	42	2018
Replicator dynamics for public goods game with resource allocation in large populations	Wang, Qiang; He, Nanrong and Chen, Xiaojie	41	2018
Doubly effects of information sharing on interdependent network reciprocity	Xia, Chengyi; Li, Xiaopeng; Wang, Zhen and Perc, Matjaž	33	2018
Aspiration-based coevolution of link weight promotes cooperation in the spatial Prisoner’s Dilemma game	Shen, Chen; Chu, Chen; Shi, Lei; Perc, Matjaž and Wang, Zhen	30	2018
Heterogeneous update mechanisms in evolutionary games: mixing innovative and imitative dynamics	Amaral, Marco Antonio and Javarone, Marco Alberto	26	2018
Evolutionary dynamics of cooperation in neutral populations	Szolnoki, Attila and Perc, Matjaž	25	2018

**Table 4.** The most central authors based on co-authorship. (We evaluate how central authors are based on different centrality measures. More specifically, we report on who is the most connected author (degree centrality) and who are closest to the rest of the nodes (closeness centrality). Also we report on the ones through which passes more information (betweenness centrality) and the ones connected to other central nodes (eigenvector centrality). Although there is some shifting between the most central authors based on different measures most of the names remain the same over all the measures. All the names are identified as males.)

name	betweenness (weighted) central	name	closeness central	name	eigenvector (weighted) central	name	degree central
Perc, Matjaž	0.181886	Wang, Zhen	0.239236	Wang, Long	0.465536	Wang, Zhen	0.028754
Wang, Zhen	0.157579	Perc, Matjaž	0.237049	Chen, Xiaojie	0.412551	Perc, Matjaž	0.024920
Nowak, Martin A.	0.112082	Moreno, Yamir	0.229596	Perc, Matjaž	0.392323	Nowak, Martin A.	0.021725
Wu, Bin	0.083079	Wu, Bin	0.228267	Szolnoki, Attila	0.367865	Traulsen, Arne	0.015122
Xia, Chengyi	0.080835	Wang, Long	0.227548	Fu, Feng	0.224587	Xia, Chengyi	0.014483
Iwasa, Yoh	0.074673	Shi, Lei	0.223284	Wu, Te	0.217658	Wang, Long	0.013419
Wang, Chao	0.068694	Szolnoki, Attila	0.222343	Wang, Zhen	0.167830	Wang, Binghong	0.013206
Traulsen, Arne	0.067122	Traulsen, Arne	0.222301	Liu, Yongkui	0.121802	Chen, Xiaojie	0.012993
Rand, David G.	0.062677	Boccaletti, Stefano	0.220630	Szabó, György	0.115295	Shi, Lei	0.011289
Zhang, H	0.059437	Xia, Chengyi	0.220205	Wu, Bin	0.109007	Tuyls, Karl	0.010224
Shi, Lei	0.058491	Nowak, Martin A.	0.220154	Zhang, Yanling	0.089163	Sanchez, Angel	0.009585
Cressman, Ross	0.056038	Chen, Xiaojie	0.219413	Nowak, Martin A.	0.088939	Li, Xiang	0.009585
Chen, Xiaojie	0.052469	Iwasa, Yoh	0.218606	Cong, Rui	0.085074	Brown, Joel S.	0.009159
Moreno, Yamir	0.051839	Rand, David G.	0.217825	Li, Zhi	0.078230	Rong, Zhihai	0.008946
Chen, Wei	0.051137	Jusup, Marko	0.217160	Wang, Jing	0.069681	Moreno, Yamir	0.008946
